# The Role of Central Adiposity in the Link Between Obstructive Sleep Apnea and Frailty: Insights From NHANES 2015-2018

**DOI:** 10.1093/geroni/igaf122.2093

**Published:** 2025-12-31

**Authors:** Ting Xu, Xichenhui Qiu, Qiqi Hang, Xiang Qi, Haokun Mei, Minghui Ji, Qin Xu, Bei Wu

**Affiliations:** Nanjing Medical University, Nanjing, Jiangsu, China (People’s Republic); Shenzhen University, Nanshan, Jiangsu, China (People’s Republic); The Second Clinical School, Nanjing Medical University, Nanjing, Jiangsu, China (People’s Republic); New York University, New York, New York, United States; The Fourth Clinical School, Nanjing Medical University, Nanjing, Jiangsu, China (People’s Republic); Nanjing Medical University, Nanjing, Jiangsu, China (People’s Republic); Nanjing Medical University, Nanjing, Jiangsu, China (People’s Republic); NYU Shanghai, Shanghai, China (People’s Republic)

## Abstract

We investigated how body fat distribution, specifically central adiposity measured by the A Body Shape Index (ABSI), modifies the association between obstructive sleep apnea (OSA) and frailty and explored racial /ethnic differences, particularly focus on non-Hispanic White individuals. We used data from 4,154 adults in the NHANES 2015–2018. Frailty was measured using the 36-item Frailty Index. We applied logistic regression models to investigate the association between OSA and frailty, grouping participants into three levels (tertiles) based on their ABSI scores, with particular focus on the role of non-Hispanic White individuals. Among the 4,154 participants, 2,376 (57.2%) had OSA, and 469 (19.7%) were classified as frail. Individuals with OSA had a 1.5 times higher likelihood of frailty (P < 0.001). However, this association varied based on central adiposity levels. In participants with the lowest ABSI, no significant relationship was found between OSA and frailty. In contrast, the association was significant among those with moderate (OR = 1.564, 95% CI: 1.062–2.303, P = 0.049) and high (OR = 1.681, 95% CI: 1.105–2.556, P = 0.038) ABSI levels, indicating that greater central adiposity amplifies the OSA-frailty link. Racial differences were also evident. Non-Hispanic White participants exhibited a stronger association between OSA and frailty (OR = 1.829, 95% CI: 1.148–2.914, P = 0.009) compared to other racial/ethnic groups in the intermediate ABSI group. These findings emphasize the role of central adiposity in frailty risk among individuals with obstructive sleep apnea and underscore the need for race/ethnicity-specific risk assessments.

